# Whole-genome comparisons of *Penicillium* spp. reveals secondary metabolic gene clusters and candidate genes associated with fungal aggressiveness during apple fruit decay

**DOI:** 10.7717/peerj.6170

**Published:** 2019-01-09

**Authors:** Guangxi Wu, Wayne M. Jurick II, Franz J. Lichtner, Hui Peng, Guohua Yin, Verneta L. Gaskins, Yanbin Yin, Sui-Sheng Hua, Kari A. Peter, Joan W. Bennett

**Affiliations:** 1 Oak Ridge Institute for Science and Education, Oak Ridge, TN, USA; 2 Food Quality Laboratory, USDA-ARS, Beltsville, MD, United States of America; 3 Department of Plant Biology, Rutgers, The State University of New Jersey—Camden, New Brunswick, NJ, United States of America; 4 Department of Biological Sciences, Northern Illinois University, DeKalb, IL, United States of America; 5 Western Regional Research Center, USDA-ARS, Albany, CA, United States of America; 6 Plant Pathology and Environmental Microbiology, Penn State University, Biglerville, PA, USA

**Keywords:** *Penicillium* spp, Blue mold, Pome fruit, Comparative genomics, Gene profiling

## Abstract

Blue mold is a postharvest rot of pomaceous fruits caused by *Penicillium expansum* and a number of other *Penicillium* species*.* The genome of the highly aggressive *P. expansum* strain R19 was re-sequenced and analyzed together with the genome of the less aggressive *P. solitum* strain RS1. Whole genome scale similarities and differences were examined. A phylogenetic analysis of *P. expansum, P. solitum,* and several closely related *Penicillium* species revealed that the two pathogens isolated from decayed apple with blue mold symptoms are not each other’s closest relatives. Among a total of 10,560 and 10,672 protein coding sequences respectively, a comparative genomics analysis revealed 41 genes in *P. expansum* R19 and 43 genes in *P. solitum* RS1 that are unique to these two species. These genes may be associated with pome fruit–fungal interactions, subsequent decay processes, and mycotoxin accumulation. An intact patulin gene cluster consisting of 15 biosynthetic genes was identified in the patulin producing *P. expansum* strain R19, while only a remnant, seven-gene cluster was identified in the patulin-deficient *P. solitum* strain*.* However, *P. solitum* contained a large number of additional secondary metabolite gene clusters, indicating that this species has the potential capacity to produce an array of known as well as not-yet-identified products of possible toxicological or biotechnological interest.

## Introduction

Postharvest “blue mold” is the most common and economically deleterious pome fruit rot worldwide (Rosenberger, 1990; Xiao & Boal, 2009). Many *Penicillium* species (*P. auarantiogriseum, P. carneum, P. commune*, *P. brevicompactum, P. crustosum, P. solitum, P. verrucosum* and *P. expansum*) cause blue mold during long-term storage of apples, pears, quince and sometimes other fruits (Raper & Thom, 1949; Sanderson & Spotts, 1995; Sholberg & Haag, 1996; Pianzzola, Moscatelli & Vero, 2004). The fungus gains access to stored fruits primarily through stem punctures/wounds and bruises. On apples, *P. expansum* is the most cosmopolitan and aggressive species (Pianzzola, Moscatelli & Vero, 2004) while *P. solitum* is less aggressive and causes significantly less decay in storage (Pitt et al., 1991).

Isolates of *P. expansum* regularly produce patulin, a regulated mycotoxin, while all tested *P. solitum* isolates are non-toxigenic (Frisvad, 2004; Jurick et al., 2010). Comparisons of the patulin biosynthetic pathway have been conducted between *P. expansum*, *P. italicum* and *P. digitatum* but not other blue mold causing fungi (Li et al., 2015). By comparing the genomes of the aggressive and toxigenic *P. expansum*, the less aggressive and nontoxigenic *P. solitum*, and other closely related *Penicillium* species, we sought to identify differences that provide insights into blue mold decay of stored pome fruits. With the aim of devising novel strategies for management of these economically important fungi, it is important to understand the genetic basis of the secondary metabolite production which is facilitated by a well sequenced and annotated genome.

*Penicillium expansum* R19 was the first *P. expansum* strain to be sequenced, published and released to the public domain (Yu et al., 2014). Although several other strains of *P. expansum* have been sequenced since that time (Li et al., 2015; Ballester et al., 2015), to the best of our knowledge, there has been no comparative genomic analysis amongst *Penicillium* species causing blue mold on pome fruit. Therefore, we conducted a detailed study comparing different species of these fungi at the genome level. Recently, we sequenced the genome of *P. solitum* strain RS1 and achieved a high-quality assembly (Yu et al., 2016). Here, we re-sequenced the highly aggressive and patulin-producing *P. expansum* strain R19 to achieve a better assembly, and then compared the two blue mold genomes to each other, as well as to the genomes of five other *Penicillium* species that inhabit different ecological niches and hosts.

Our analysis included the genomes of two citrus pathogens, *Penicillium digitatum* (“pdi”) (Marcet-Houben et al., 2012) and *Penicillium italicum* (“pit”) (Ballester et al., 2015); two cheese making species, *Penicillium camemberti* (“pca”) and *Penicillium roqueforti* (“pro”) (Cheeseman et al., 2014); and the penicillin-producing industrial strain *Penicillium chrysogenum* (“pch”) (Van den Berg et al., 2008). We report differences and similarities in the gene repertoires of *P. expansum* and *P. solitum*, and discuss the potential functions of some of their unique genes. A robust phylogenetic analysis was performed using nearly four hundred core eukaryotic genes (CEGs) to compare the two blue mold species to the five other *Penicillium* genomes. Our data provides a solid platform to further explore the genetic basis of the differences in the ability of *P. expansum* and *P. solitum* to cause postharvest decay of pome fruits, uncover new insights into secondary metabolic gene clusters, and strengthen our understanding of genome-wide phylogenetic relationships amongst economically important *Penicillium* species.

## Materials and Methods

### Fungal growth, genomic DNA extraction, genome sequencing, assembly, and annotation

Growth, both in culture and *in vivo* including inoculation of apple*,* and genomic DNA extraction of *P. expansum* R19 were conducted using the same approaches as previously published (Conway, 1982; Yu et al., 2014). The sequencing, assembly, and annotation pipeline previously described in our study on *P. solitum* RS1 (Yu et al., 2016) was used for the genome of *P. expansum* R19. Briefly, the assembly was conducted using HGAP3 under default settings, which applies long read correction algorithms and the Celera assembler to confidently produce high quality contigs (unitigs) using reads from PacBio Single Molecule Real Time (SMRT) sequencing technology (Ricker et al., 2016). The annotation software MAKER (Holt & Yandell, 2011) was implemented for four iterative runs starting with gene predictions from CEGMA (Parra, Bradnam & Korf, 2007). This newly sequenced *P. expansum* genome was 32,356,049 bp which is 97.3% of the currently published *P. expansum* MD-8 genome. The BUSCO (v. 3.0.2) analysis resulted in 289 complete BUSCOs out of 290 (dikarya odb9) and one fragmented BUSCO for a 99.7% complete assembly (Waterhouse et al., 2018). In the annotation step, *P. solitum* proteins were used as a part of the protein evidence set instead of *P. expansum* proteins.

### *Penicillium* species phylogenomics

Whole genome phylogeny of seven *Penicillium* species and one *Aspergillus flavus* strain was built using peptide sequences of shared core eukaryotic genes (CEGs) as predicted by CEGMA (Parra, Bradnam & Korf, 2007). The source data for the other included genomes came from NCBI and EMBL, *A. flavus* NRRL 3357 (https://www.ncbi.nlm.nih.gov/nuccore/EQ963472), *P. digitatum* Pd1(http://fungi.ensembl.org/Penicillium_digitatum_pd1_gca_000315645/Info/Index),*P. camemberti* FM 013 (https://fungi.ensembl.org/Penicillium_camemberti_fm_013_gca_000513335/Info/Index), *P. chrysogenum* Wisconsin 54-1255 (http://fungi.ensembl.org/Penicillium_rubens_wisconsin_54_1255_gca_000226395/Info/Index), *P. italicum* PHI-1 (https://genome.jgi.doe.gov/Penita1/Penita1.home.html), and *P. roqueforti* FM 164 (https://fungi.ensembl.org/Penicillium_roqueforti_fm164_gca_000513255/Info/Index)*.* We used a set of 399 CEGs that satisfy the following criteria: (1) They are present in all eight above-mentioned genomes; (2) all sequences are at least 90% the length of their *Saccharomyces cerevisiae* orthologs (Engel et al., 2014). Sequences were first aligned with MAFFT with parameters—localpair—maxiterate 1,000 (Katoh et al., 2002), and then concatenated. The phylogeny was inferred using RAxML (Stamatakis, 2006) with the parameters “–f a -# 400 –m PROTGAMMAJTT”.

### Markov cluster algorithm (MCL) clustering

The protein sequences of the seven selected *Penicillium* species were subjected to BLAST comparison against each other (Altschul et al., 1990). The bitscores were then used to generate clusters of genes via the TRIBE-MCL algorithm (Enright, Van Dongen & Ouzounis, 2002) using default settings.

### Gene Ontology (GO) annotation and Fisher’s exact tests

*P. expansum* R19 protein sequences were used in a BLAST comparison against the NCBI NR protein database and the results were used to predict GO annotation using Blast2go with default settings (http://www.blast2go.com). Further, GO enrichment in selected groups of genes was tested using Fisher’s exact tests. The same analysis was also performed on *P. solitum* RS1 protein sequences.

### Secondary metabolite gene clusters

Secondary metabolite gene clusters were identified using the antiSMASH program for fungi at default settings (Weber et al., 2015).

## Results

**Figure 1:**
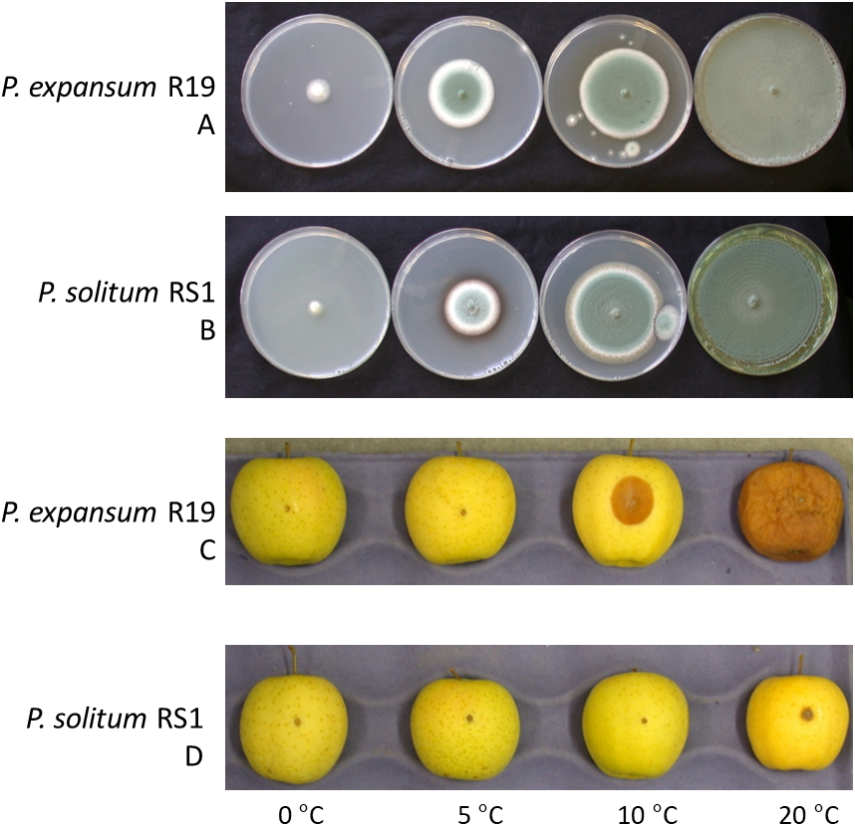
Effect of temperature on fungal growth in culture and during apple fruit decay. (A) *Penicillium expansum* growing on Potato Dextrose Agar. (B) *Penicillium solitum* growing on Potato Dextrose Agar. (C) Penicillium expansum causing blue mold decay on apple fruit. (D) *Penicillium solitum* causing blue mold decay on apple fruit.

The growth of *Penicillium expansum* and *P. solitum* was compared, both on agar plates as well as the aggressiveness on apples incubated at different temperatures. When cultured on potato dextrose agar, both fungi grew on all temperatures ranging from 0 to 20 °C although *P. solitum* grew slightly slower than *P. expansum* (Figs. 1A, 1B). However, when inoculated onto apple fruit, the differences in aggressiveness were much more pronounced (Figs. 1C, 1D)*.* Both pathogens are necrotrophic and require a wound for infection, they are unable to puncture the skin of the fruit due to the lack of an appressorium and rely on pectin degrading enzyme production for growth (Yao, Conway & Sams, 1996; Jurick et al., 2009, p. 20019). Decay was evident 7 days post inoculation (DPI) on apples inoculated with *P. expansum* at 10 and 20 °C, while the apples inoculated with *P. solitum* displayed only a small lesion around the inoculation point at 20 °C (Figs. 1C, 1D).

**Figure 2:**
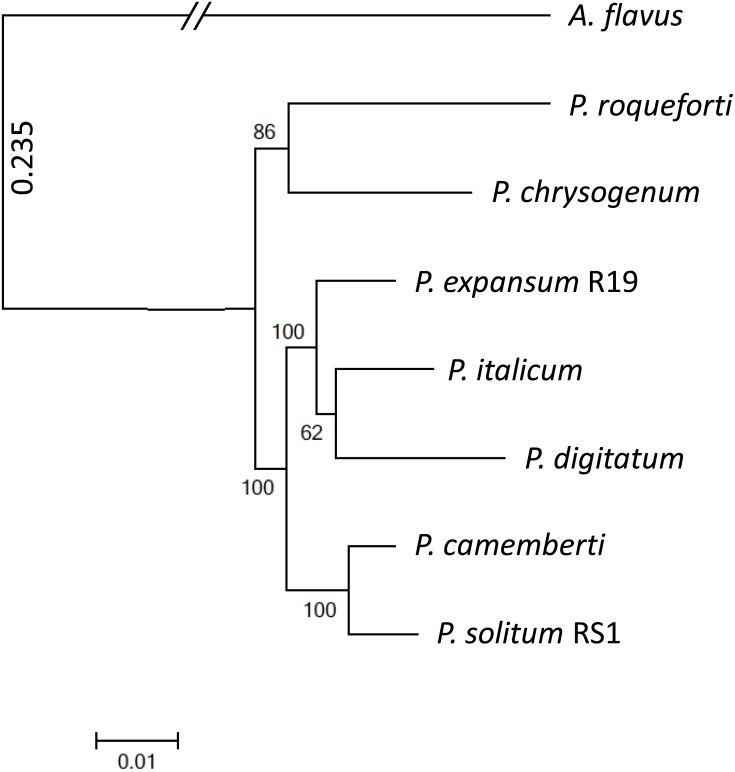
Phylogenomic analysis of seven *Penicillium* species. *A. flavus* was used as outgroup and bootstrap values are indicated on the branches.

To achieve optimal genome sequence and ensure reliable downstream bioinformatic comparisons, the PacBio Single Molecule Real Time (SMRT) sequencing platform was utilized to resequence *P. expansum* R19, with sequencing coverage reaching 65-fold. An assembly of 16 unitigs with an N50 value of 8.17 Mbp was achieved and the genome assembly was deposited under BioSample SUB4649056. Gene annotation revealed 10,560 putative protein coding genes. Using CEMGA 99.2% complete core eukaryotic genes (CEGs) and 99.6% partial (including complete) CEGs were predicted in the genome. A genome-wide multi-gene phylogenetic approach was then implemented, involving 399 CEGS, using *P. expansum*, *P. solitum*, and several other related *Penicillium* species, with *Aspergillus flavus* as a designated outgroup (Fig. 2). The closest relative of *P. solitum* amongst the *Penicillium* spp. was *P. camemberti*, while for *P. expansum,* the most phylogenetically similar were the two citrus pathogens *P. digitatum* and *P. italicum* (Fig. 2). *P. roqueforti*, a cheese making species associated with blue cheeses and *P. chrysogenum*, the penicillin-producing species, formed another distant, yet distinct clade (Fig. 2).

For *P. expansum* R19, 7,921 proteins were annotated (75% of all proteins) with a total of 37,962 GO entries (4.8 GO entries per protein), representing 6,000 unique GO categories. For *P. solitum* RS1, 7,920 proteins were annotated (74.2% of all proteins) with a total of 37,858 GO entries (4.8 GO entries per protein), representing 5,993 unique GO categories. We analyzed GO enrichment in a group of 877 proteins that were over-represented in *P. expansum*, defined as having ≥ 2X more copies than detected in *P. solitum* (Table 1). We also tested the GO enrichment in 550 *P. expansum* proteins that were over-represented in both blue mold fungi, defined as having on average ≥ 2X copies in *P. expansum* and *P. solitum* than in the five *Penicillium* species included in this study (Table 2). An analysis using *P. solitum* proteins over-represented in the two pome blue mold *Penicillium* species revealed similar results. Also, we examined the GO enrichment in a group of 1,096 proteins that are represented more frequently in *P. solitum*, defined as having ≥ 2X copies in *P. solitum* than in *P. expansum* (Table 3). The seven *Penicillium* species included in this study, with a total of 9,960 protein families, encompassing 78,479 proteins, were identified. All species shared 5,308 protein families, encompassing 64,598 proteins, or 82.3% of the total proteins also referred to as the proteome. For *P. expansum* 86.4% (9,119 out of 10,560), and for *P. solitum* 85.3% (9,099 out of 10,672) of the proteins were shared with the other species. However, a small set (36) of protein families were unique to the two blue mold species isolated from pome fruits, accounting for 41 proteins in *P. expansum* and 43 proteins in *P. solitum* (Table S1). Amino acid sequences from both fungi that cause blue mold of pome fruits were also compared. The more aggressive species, *P. expansum* R19, contained 222 gene families, encompassing 261 proteins that were not present in the less aggressive species *P. solitum* RS1. Similarly, there were 299 gene families, accounting for 375 proteins present in *P. solitum* but not in *P. expansum*, (Table S1). In addition, *P. expansum* R19 contained significant numbers of proteins with copy numbers that are one to several fold more than *P. solitum* RS1 (Table S1).

**Table 1:** GO enrichment tests of over-represented *P. expansum* proteins.

GO-ID	Term	Type	FDR	*P*-Value	SG	NSG	SNG	NSNG
GO:0003824	catalytic activity	F	2.37E–05	2.80E–09	319	3,992	155	3,455
GO:0016829	lyase activity	F	1.59E–03	3.75E–07	37	218	437	7,229
GO:0008152	metabolic process	P	1.81E–03	6.39E–07	369	5,015	105	2,432
GO:0046271	phenylpropanoid catabolic process	P	1.34E–02	1.27E–05	4	0	470	7,447
GO:0046281	cinnamic acid catabolic process	P	1.34E–02	1.27E–05	4	0	470	7,447
GO:0034396	negative regulation of transcription from RNA polymerase II promoter in response to iron	P	1.34E–02	1.27E–05	4	0	470	7,447
GO:0034395	regulation of transcription from RNA polymerase II promoter in response to iron	P	1.34E–02	1.27E–05	4	0	470	7,447
GO:0009803	cinnamic acid metabolic process	P	1.34E–02	1.27E–05	4	0	470	7,447
GO:0019748	secondary metabolic process	P	3.10E–02	3.29E–05	21	111	453	7,336
GO:0016813	hydrolase activity, acting on carbon-nitrogen (but not peptide) bonds, in linear amidines	F	3.93E–02	5.32E–05	6	7	468	7,440
GO:0071281	cellular response to iron ion	P	3.93E–02	6.03E–05	4	1	470	7,446
GO:0010039	response to iron ion	P	3.93E–02	6.03E–05	4	1	470	7,446
GO:0009698	phenylpropanoid metabolic process	P	3.93E–02	6.03E–05	4	1	470	7,446
GO:0071241	cellular response to inorganic substance	P	4.27E–02	7.05E–05	7	12	467	7,435

**Notes.**

Type indicates the category of GO terms (P: biological process; F: molecular function).

FDR False Discovery Rate SG the number of proteins that are in the tested group and have the GO term NSG the number of proteins that are not in the tested group, and have the GO term SNG the number of proteins that are in the tested group, and do not have the GO term NSNG the number of proteins that are not in the tested group, and do not have the GO term

**Table 2:** GO enrichment tests of *P. expansum* proteins over-represented in the two pome fruit decaying *Penicillium* species.

GO-ID	Term	Type	FDR	*P*-Value	SG	NSG	SNG	NSNG
GO:0009812	flavonoid metabolic process	P	3.48E–03	4.11E–07	4	0	198	7,719
GO:0047661	amino-acid racemase activity	F	8.53E–03	2.01E–06	4	1	198	7,718
GO:0036361	racemase activity, acting on amino acids and derivatives	F	1.25E–02	5.92E–06	4	2	198	7,717
GO:0016855	racemase and epimerase activity, acting on amino acids and derivatives	F	1.25E–02	5.92E–06	4	2	198	7,717
GO:0010333	terpene synthase activity	F	4.49E–02	2.65E–05	4	4	198	7,715

**Notes.**

Type indicates the category of GO terms (P: biological process; F: molecular function).

FDR False Discovery Rate SG the number of proteins that are in the tested group and have the GO term NSG the number of proteins that are not in the tested group, and have the GO term SNG the number of proteins that are in the tested group, and do not have the GO term NSNG the number of proteins that are not in the tested group, and do not have the GO term

**Figure 3:**
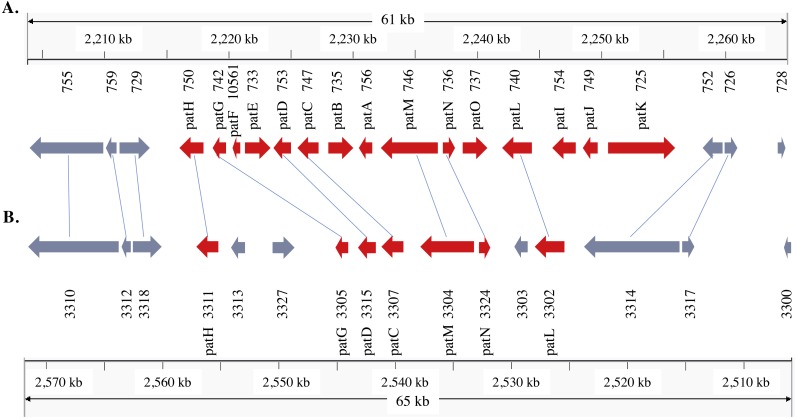
Patulin gene cluster in *Penicillium expansum* (A) and *P. solitum* (B). Gene IDs and gene names are indicated below the genes. Reciprocal best matches are linked with a line. Red colored genes are known members of the patulin gene cluster.

**Table 3:** GO enrichment tests of over-represented *P. solitum* proteins.

GO-ID	Term	Type	FDR	*P*-Value	SG	NSG	SNG	NSNG
GO:0046983	protein dimerization activity	F	1.49E–09	1.77E–13	29	63	478	7,350
GO:0042559	pteridine-containing compound biosynthetic process	P	1.23E–04	2.91E–08	10	8	497	7,405
GO:0006729	tetrahydrobiopterin biosynthetic process	P	7.47E–04	4.43E–07	6	1	501	7,412
GO:0046146	tetrahydrobiopterin metabolic process	P	7.47E–04	4.43E–07	6	1	501	7,412
GO:0008124	4-alpha-hydroxytetrahydrobiopterin dehydratase activity	F	7.47E–04	4.43E–07	6	1	501	7,412
GO:0042558	pteridine-containing compound metabolic process	P	1.29E–03	9.17E–07	10	14	497	7,399
GO:0015074	DNA integration	P	5.73E–03	4.76E–06	6	3	501	7,410
GO:0046654	tetrahydrofolate biosynthetic process	P	1.56E–02	1.66E–05	4	0	503	7,413
GO:0003934	GTP cyclohydrolase I activity	F	1.56E–02	1.66E–05	4	0	503	7,413
GO:0016829	lyase activity	F	1.99E–02	2.36E–05	35	227	472	7,186
GO:0000981	RNA polymerase II transcription factor activity, sequence-specific DNA binding	F	2.12E–02	2.76E–05	41	289	466	7,124

**Notes.**

Type indicates the category of GO terms (P: biological process; F: molecular function).

FDR False Discovery Rate SG the number of proteins that are in the tested group and have the GO term NSG the number of proteins that are not in the tested group, and have the GO term SNG the number of proteins that are in the tested group, and do not have the GO term NSNG the number of proteins that are not in the tested group, and do not have the GO term

**Figure 4:**
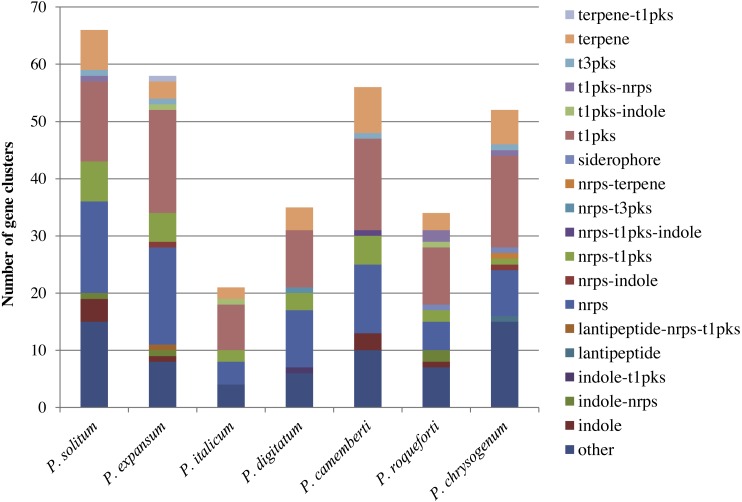
Composition of secondary metabolism gene clusters and their backbone genes in seven different *Penicillium* species. SM gene cluster types in seven different* Penicillium* spp.

In the *P. expansum* R19 genome, we identified the fifteen-gene patulin cluster (Fig. 3). In the *P*. *solitum* genome, a sequence similarity search (BLAST) identified a partial patulin gene cluster, spanning 31 kb (Fig. 3). Within the *P. solitum* partial gene cluster, only seven of the identified patulin biosynthetic genes were present (Fig. 3), with amino acid sequence identity >91% and *e*-value = 0. The seven genes identified from the partial cluster were *patC*, *patD*, *patG*, *patH*, *patL*, *patM*, and *patN* (Fig. 3). Five other genes (*patA*, *patB*, *patE*, *patK*, and *patO*) were found dispersed elsewhere in the genome with much lower amino acid sequence identity (between 29% and 49%). Three genes, *patF*, *patI* and *patJ*, were not found anywhere in the genome (*e*-value cutoff of 1e^−5^). Using the antiSMASH platform, all seven of the *Penicillium* spp. genomes were analyzed to determine their secondary metabolism genes. Within the *P. solitum* genome, we identified 66 gene clusters that were putatively involved in secondary metabolism (SM), more than any other of the *Penicillium* species analyzed in this study (Fig. 4). In contrast, 58 gene clusters were found in *P. expansum*, 21 gene clusters in *P. italicum,* and 35 gene clusters in *P. digitatum*.

## Discussion

The first aim of this study was to provide a high quality, annotated, and assembled genome sequence of *P. expansum* (R19), so that it could be used for further downstream bioinformatic analyses. The longer reads achieved by the PacBio platform enabled better assembly and resulted in fewer gaps than illumina sequencing has produced. PacBio SMRT sequencing technology allows for Hence, an assembly of 16 unitigs with an N50 value of 8.17 Mbp was achieved, a major improvement over the 48.5 kbp previously achieved using Illumina (Yu et al., 2014).

Although *P. expansum* and *P. solitum* cause blue mold decay of pome fruits (Frisvad, 2004; Jurick et al., 2010), the closest relative of *P. solitum* was determined to be *P. camemberti*, a species named after the distinctive soft cheese with a white rind (Cheeseman et al., 2014). In addition to causing blue mold on apple, *P. solitum* has been isolated from spoiled processed meats, cheeses, and margarine which are commonly stored at 4 °C like apples (Pitt et al., 1991; Hocking et al., 1998). Moreover, *P. solitum* has been isolated from several very cold, high salt environments and has been termed an “extremophile” (Stierle et al., 2012). In contrast, for *P. expansum,* the closest relatives in our analyses were *P. digitatum* (Marcet-Houben et al., 2012) and *P. italicum* (Ballester et al., 2015) indicating that ancestors of these three species may have occupied the same carbohydrate-rich niches associated with fruit decay.

**Table 4:** Eukaryotes that contain SnoaL-like polyketide cyclase domain (PF07366) Species name, number of sequences, group and ecological habitat containing SnoaL-like domains.

Species	Nr. of sequences	Group	Habitat
*Amphimedon queenslandica*	1	Sponge	Aquatic
*Thelohanellus kitauei*	1	Myxozoa	Aquatic/Parasitic
*Thalassiosira pseudonana*	1	Diatom	Aquatic
*Chlamydomonas reinhardtii*	1	Green algae	Aquatic
*Volvox carteri*	2	Green algae	Aquatic
*Chlorella variabilis*	1	Green algae	Aquatic
*Ostreococcus tauri*	1	Green algae	Aquatic
*Auxenochlorella protothecoides*	2	Green algae	Aquatic
*Phytophthora sojae*	1	Oomycetes	Plant pathogenic
*Phytophthora ramorum*	1	Oomycetes	Plant pathogenic
*Fusarium graminearum*	4	Ascomycetes	Plant pathogenic
*Colletotrichum sublineola*	2	Ascomycetes	Plant pathogenic
*Penicillium expansum*	1	Ascomycetes	Plant pathogenic
*Penicillium solitum*	1	Ascomycetes	Plant pathogenic
*Setaria italic*	2	Higher plants	Soil
*Triticum aestivum*	2	Higher plants	Soil

By comparing the two blue mold fungi at the whole-genome scale, our aim was to identify differences in genetic factors that are linked to pathogen aggressiveness during pome fruit rot in addition to finding genes specific to the apple fruit pathogens. Thirty-six protein families were present only in the two pome fruit pathogens (*P. expansum* and *P. solitum)*, but absent in the other five *Penicillium* species examined.. In *P. expansum* there were 222 gene families not found in *P. solitum*. of which 44 protein families were present only in *P. expansum* R19, but absent in the other species analyzed in this study. These protein families are of primary interest since they may represent specific genes involved in pome fruit maceration or account for *P. expansum*’s increased aggressiveness during apple fruit decay. Here, we discuss the potential role of several *P. expansum* and *P. solitum*-specific enzymes.

To examine similarities and differences in gene repertoires amongst the seven *Penicillium* species, we identified protein families using a Markov cluster algorithm called TRIBE-MCL that detects and categorizes eukaryotic protein families (Enright, Van Dongen & Ouzounis, 2002). Gene ontology (GO) enrichment analyses were performed using annotated protein sequences via Blast2go (Conesa et al., 2005). Several categories related to combating plant defense mechanisms were enriched, including phenylpropanoid catabolic process, cinnamic acid catabolism, and response to iron ion sequestration (siderophores). Flavonoid metabolism, a specific gene category involved in plant defense, was enriched, which may be beneficial for the fungus to overcome the phenolic-rich (quercetin) environment of the apple host tissue, known to be important in basal defense systems of apple fruits (Sun et al., 2017). These categories included lyase activity. It is well established that lyases degrade cell wall components like pectin and they have been shown to be involved in the maceration of pome fruit tissues by *P. expansum* and *P. solitum* (Yao, Conway & Sams, 1996; Jurick et al., 2009).

Single copies of SnoaL-like polyketide cyclases (PF07366) are found in both *P. expansum* and *P. solitum* (Table S1). A more detailed analysis of PF07366 on http://pfam.xfam.org/ reveals their presence in a number of prokaryotes, in two other fungal species, and a few other eukaryotes (Table 4). These eukaryotic members are either aquatic or plant related (Table 4), suggesting that this gene might have been laterally transferred between organisms living in close proximity or occupying the same ecological niche. These cyclases are backbone genes for polyketide secondary metabolite biosynthesis and thus involved in fungal secondary metabolism. A Glucose-6-phosphate isomerase (PF10432) is found in a number of prokaryotes and only two eukaryotes, *P. expansum* and *P. solitum* (http://pfam.xfam.org/, Table S1), suggesting that this gene might have been laterally transferred. The exact function it plays in pome fruit decay warrants further exploration via RNAi and /or gene deletion studies.

A thermolysin metallopeptidase (PF01447) was found only in *P. expansum*, but not in *P. solitum* or any of the other five *Penicillium* species included in this study. Peptidases are known virulence factors for fungal plant pathogens such as *Fusarium graminearum*. They break down plant host proteins to provide nutrients for fungal growth, reproduction, and colonization (Lowe et al., 2015). This *P. expansum* unique metallopeptidase may play a role in pome fruit decay and should be explored further. In addition to the above-mentioned examples, several putative transcription factors, hydrolases, and other enzymes that are unique to one or both blue mold *Penicillium* species were uncovered (Table S1). They provide a guide for future research to pinpoint the genes essential for postharvest apple fruit decay.

Patulin is a well-studied, polyketide-derived mycotoxin commonly found in apple products. Due to its carcinogenic effects, the European Union (EU) has set limits on the maximum allowable amount of patulin to 50 µg/L for juice and fruit derived products, 25 µg/L for solid apple products, and 10 µg/L for juices and food for babies and infants (European Union, 2003). The U.S. Food and Drug Administration (FDA), as well as regulatory agencies in other countries, have imposed limits on the permissible amounts of patulin in fruit juices and processed pome fruit products for human consumption at 50µg/L (Puel, Galtier & Oswald, 2010). There are 15 genes involved in patulin biosynthesis found within a well-defined gene cluster (Tannous et al., 2014; Li et al., 2015; Ballester et al., 2015). The structural organization of the cluster is shown in the same order in our *P. expansum* R19 strain as reported earlier (Tannous et al., 2014). The partial patulin gene cluster arrangement in *P. solitum* was very similar to that found in *P. camemberti* (Ballester et al., 2015), with the same seven patulin biosynthetic genes present and arranged in the same configuration. The incomplete patulin gene cluster found in both *P. solitum* and in *P. camemberti* may explain why these species are unable to produce patulin in either liquid or solid culture conditions (Frisvad, 2004). Curiously, some of the patulin genes were dispersed outside of the cluster, likely due to chromosomal rearrangements. Our genomic data suggests that the ability to produce patulin was lost in the ancestor of these two species.

While patulin is the mycotoxin of primary concern to apple producers, processors and packers in the U.S. and EU, it is only one of many secondary metabolites known to be produced by *Penicillium* species (Puel, Galtier & Oswald, 2010; Wright, 2015). Genes responsible for the synthesis of secondary metabolites are generally organized together in clusters in fungal genomes. Despite the large number of putative SM gene clusters in these *Penicillium* species, only a few gene products such as patulin, penicillic acid, and citrinin are well studied in agriculture and food science. In addition, because *P. solitum* is an extremophile, there are reports of several interesting secondary metabolites isolated from unusual environments that merit further study in order to determine if they are produced in apples with blue mold symptoms. For example, a strain of *P. solitum* isolated from The Berkeley Pit, a former copper mine located in Butte, Montana produces two drimane sesquiterpene lactones named Berkedrimanes A and B (Stierle et al., 2012). These secondary metabolites inhibit the signal transduction enzymes caspase-1 and caspase-3 and mitigate the production of interleukin 1- β in a leukemia cell line (Stierle et al., 2012). Another biologically active metabolite from *P. solitum* is solistatin, a phenolic compactin analogue related to Mevastatin, one of the classes of statins which inhibit HMG-CoA reductase, which are widely prescribed as cholesterol lowering agents (Sørensen et al., 1999).

Across the fungal kingdom, however, the products of bioinformatically discovered SM gene clusters are largely unknown. Some clusters are functional and expressed only under specific conditions (e.g., during competition for nutrients or stress conditions), but not expressed under routine conditions in the laboratory (Tannous et al., 2014; Li et al., 2015). In light of our recent findings, it remains unexplored whether the large number of bioinformatically detected SM gene clusters in the *P. solitum* genome translates into a greater capacity for producing various secondary metabolites on apple, and if any of these metabolites might pose hitherto undiscovered risks to human health. Further characterization of these identified gene clusters, via functional gene deletion studies, will help ascertain our understanding of SM gene cluster function and the mechanisms of secondary metabolism/biosynthesis.

## Conclusions

Comparison of the genomes of *P. expansum* and *P. solitum* has provided a unique opportunity to explore the genetic basis of the differential ability of these two species to cause blue mold decay. Key genes identified here can now be analyzed functionally to confirm their involvement in apple fruit decay. Fungal gene networks, pathways, and regulators can be exploited to design specific control strategies to block decay. Additionally, our comparative genomic data has clarified phylogenetic relationships between *Penicillium* species that occupy different ecological niches. Additionally, the SM gene cluster repertoire has been elucidated in both *P. expansum* and *P. solitum* and provided evidence to explain the lack of patulin production in *P. solitum* (RS1), while also revealing the potential for discovery of hitherto unknown secondary metabolites of possible biotechnological use. In agriculture, these genomic findings will guide apple fruit producers to help maintain safe, high quality apples during long-term storage, as well as being of interest to the food processing industry, plant pathologists, and the broader scientific community.

##  Supplemental Information

10.7717/peerj.6170/supp-1 Table S1 Gene clusters in *P. expansum* (R19) and *P. solitum* (RS1) Gene cluster identities in* Penicillium expansum* and* P. solitum*.

## Additional Information and Declarations

### Data Availability

The following information was supplied regarding data availability: We have presented raw data as Table S1. The r19 genome sequence data can be found in GenBank (SAMN02716838).
